# Communication barriers for d/Deaf people with cancer: a scoping review

**DOI:** 10.1007/s00520-026-11202-y

**Published:** 2026-09-09

**Authors:** Niomie Wogan, Ruth Habibi, Lesley Fallowfield, Helena Harder

**Affiliations:** Sussex Health Outcomes Research and Education in Cancer (SHORE-C), Brighton and Sussex Medical School, Universities of Brighton and Sussex, Falmer, Brighton, UK

**Keywords:** d/Deaf, Cancer, Communication, Barriers, Healthcare professionals, Communication support professionals

## Abstract

**Purpose:**

Significant healthcare disparities can be experienced by d/Deaf individuals, particularly in accessing effective communication and health information. Limited research has examined the experiences of d/Deaf cancer patients or how their interactions with healthcare professionals (HCPs) and communication support professionals (CSPs) influence their quality of care. This scoping review aimed to map current evidence about the communication and unmet needs of d/Deaf cancer patients, educational needs of HCPs and roles of CSPs.

**Methods:**

Following the Joanna Briggs Institute method for Scoping Reviews, database searches were conducted on PsycInfo, PubMed, Embase, CINAHL and Scopus, and grey literature was identified. Papers published in English that reported on the experiences of d/Deaf cancer patients and their communication with HCPs and CSPs were included. Thematic analysis was used to identify key descriptive themes.

**Results:**

Only nine (1 quantitative, 7 qualitative, 1 mixed-methods) papers were included. Most focused on d/Deaf cancer patients (mainly breast cancer); only 1 included HCPs and none examined CSPs. Three main themes and 9 subthemes were identified that negatively impacted d/Deaf cancer patients’ views about their healthcare: system-level factors; interpersonal factors and patient-level factors. The HCP-focused paper highlighted that collaboration with interpreting services enhanced communication and patient understanding.

**Conclusions:**

Despite limited data, this review highlights multiple barriers d/Deaf patients can encounter when accessing cancer services. Collectively, these may affect effective communication practices and can compromise the quality of care delivered. Data supports accessible educational resources, improved access to qualified medical interpreters and deaf awareness training for HCPs.

**Supplementary Information:**

The online version contains supplementary material available at https://doi.org/10.1007/s00520-026-11202-y.

## Introduction

The World Health Organisation estimates that there are 430 million people worldwide who are deaf or hard-of-hearing (HOH) [1]. This refers to those who have varying degrees of hearing loss and primarily use spoken language. In contrast, the World Federation of the Deaf estimates that there are over 70 million people who identify with the social model of Deaf (capital D), referring to those who are typically born deaf and feel culturally and linguistically part of the Deaf community. These individuals often communicate through sign language and may work with communication support professionals (CSPs) such as sign language interpreters [2]. In the UK, the Royal National Institute for Deaf People suggests using the term CSPs to describe the range of interpreters who facilitate communication between d/Deaf individuals and hearing professionals. This includes sign language interpreters (SLIs), lip speakers and notetakers [3] who have varying degrees of qualifications. In this scoping review, the term d/Deaf includes people who are born deaf, culturally Deaf or HOH.

Research consistently shows health inequalities within the d/Deaf population [4]. Health literacy is also lower compared to the general population [5–7], often due to lack of relevant accessible health care resources with cancer-specific information [8]. Opportunities for incidental learning, acquired through informal conversations, media and environmental cues, are less likely to occur among d/Deaf individuals, further impacting literacy development [9]. Additionally, many healthcare professionals (HCPs) rely on written communication methods with d/Deaf patients as an alternative to speech [10] which is often ineffective.

The lack of clear communication can lead to misinterpretation of medical instructions and treatment plans, increasing the risk of mismanagement and adverse health outcomes [6, 11]. Although many HCPs understand the value of CSPs, few consistently schedule them for consultations [12]. Even when a CSP is present, communication challenges may persist [13] and continuity of care is disrupted when patients do not have access to a consistent and designated interpreter [14]. As a result, many d/Deaf patients experience frustration, mistrust and, in some cases, avoidance of medical care leading to delays in diagnosis and treatment. For example, evidence shows that 64% of d/Deaf patients with prostate cancer are diagnosed at advanced or metastatic stage [15] compared with 13% in the general population [16]. Similarly, 50% of d/Deaf men lacked knowledge of prostate cancer, and 37% reported receiving no information about early detection or prevention [17].

Comparable gaps in knowledge about screening practices among d/Deaf women were also observed. A survey of 209 d/Deaf women found that 35.8% had misconceptions about the purpose of mammograms [18]. Evidence about breast cancer screening uptake was inconclusive. A US study of 1410 women reported comparable uptake of 76% among d/Deaf women compared to 82% with hearing women, with no statistically significant disparity after analysis [19]. In contrast, a large UK study of 473,185 women comparing uptake of breast and/or bowel cancer screening among people with or without recognised disabilities (including d/Deaf, n = 13,229) reported that women with disabilities were less likely than other women to participate in breast cancer screening (RR = 0.64, 95% CI, 0.62–0.65) and in bowel cancer screening (RR = 0.75, 0.73–0.76) [20]. One way to help overcome some of these healthcare barriers is through the use of CSPs such as qualified SLIs or lip speakers, which enables clear and effective communication [21]. However, many d/Deaf patients do not have access to an interpreter, or they are assisted by someone with inadequate qualifications [22]. Interacting with deaf-aware staff is associated with positive healthcare experiences, whereas a lack of understanding often leads to mistrust and discourages future healthcare-seeking [23].

Two systematic reviews involving d/Deaf patients and HCPs have examined communication support or modes of communication [24, 25]. One review found that gesturing was the most used method, followed by writing or speaking [24]. The other highlighted ineffective doctor-patient interactions due to limited cultural awareness among HCPs and misunderstandings about patients preferred communication methods [25]. While these reviews addressed general communication challenges for d/Deaf patients and provided insights on the availability of communication support, they did not explore the unique difficulties faced by d/Deaf cancer patients. For example, cancer patients often experience heightened anxiety at the point of diagnosis [26] which can restrict information recall [27]. Medical consultations, especially with cancer patients, can also be challenging, requiring patients to navigate medical jargon and technical explanations that are difficult to follow [28, 29]. A scoping review was chosen to enable a broader exploration and mapping of the available evidence [30–32] on the communication experiences of d/Deaf cancer patients, as well as the unmet educational needs of HCPs and CSPs.

## Methods

The scoping review was conducted in accordance with the framework provided by Joanna Briggs Institute methodology for scoping reviews [33], which involved (1) identifying the research question; (2) identifying relevant studies; (3) study selection; (4) charting the data and (5) collating, summarising and reporting the results. The findings were documented using the Preferred Reporting Items for Systematic Reviews and Meta-Analyses: extension for Scoping Reviews checklist (PRISMA-ScR) flow diagram [30]. The protocol was registered on the Open Science Framework (https://doi.org/10.17605/OSF.IO/9BJMG).

### Stage 1: Identifying the research question

Initial searches revealed no pre-existing scoping reviews on the topic of communication barriers experienced by d/Deaf cancer patients, or HCPs and CSPs’ experiences interacting with these patients. The review drew on qualitative and quantitative research, and the PCC framework (Population, Concept and Context) was employed (Table 1). The following research questions were used: (i) What are d/Deaf cancer patients’ communication experiences and unmet needs in healthcare settings? (ii) What are the experiences and educational needs of HCPs working with d/Deaf cancer patients? (iii) What are the experiences and educational needs of CSPs working with d/Deaf cancer patients in healthcare settings?

**Table 1:** PCC framework

Population	Studies involving the d/Deaf cancer patients, HCPs involved in the care for d/Deaf cancer patients and professionals providing communication support for d/Deaf cancer patients
Concept	Studies that focus on communication between d/Deaf people, HCPs and CSPs
Context	Any geographical location, describing all healthcare settings

### Stage 2: Identifying relevant studies

A comprehensive database search was conducted on PsycInfo, PubMed, Embase, CINAHL and Scopus. The search keywords of “deaf,” “cancer,” “healthcare professional” and “sign language interpreter” were combined using Boolean operator “OR” and between concept terms combined with “AND,” and synonyms for each keyword were used (see Supplement I). A health science subject librarian assisted in the search strategy process. Searches for grey literature were conducted on TRIP Medical Database, Google Scholar, ProQuest and Open Access Theses and Dissertations, and targeted cancer charity websites within different countries. There were no limitations regarding date of publication. Papers were hand searched and cross-referenced.

### Stage 3: Study selection

All papers were imported into EndNote21, duplicates were removed and the remainder were exported to the web-based management system Rayyan (http://rayyan.qcri.org) [34]. Two independent reviewers (NW, HH) screened the titles and abstracts, followed by a full-text review using the eligibility criteria. Any conflicts were resolved by consensus. Studies were included if they focused on d/Deaf cancer patients aged 18 years and above, HCPs providing care for d/Deaf cancer patients or CSPs such as SLIs, lip speakers, notetakers and speech-to-text reporters involved in their consultations. Eligible studies reported on communication or healthcare experiences in this context. Publications in English, spanning from database inception to 2025, employing qualitative, quantitative, or mixed-methods designs were eligible for inclusion. Both peer-reviewed and non-peer-reviewed outputs were considered, including conference abstracts, case studies, editorials and opinion pieces, were included. Studies were excluded if they did not address communication, or if they were review papers, study protocols or journal letters.

### Stage 4: Charting the data

The data extraction was performed by one author (NW) and checked by a second author (HH), and included author, year of publication, country, aim, study design, population, method to obtain data, key findings and unmet needs identified. The quality of the included papers was not appraised in this scoping review.

### Stage 5: Collating, summarising and reporting the results

The quantitative data emerging from the included studies was summarised using descriptive statistics. Qualitative data was synthesised by three authors (NW, HH, RH) using thematic analysis as outlined by Thomas and Harden’s framework [35]. This process involved line-by-line coding of the data, development of descriptive themes and generation of analytical themes. Papers were grouped accordingly, and findings were organised by theme. This stage was informed by the research team’s psychosocial-oncology background.

## Results

**Fig. 1:**
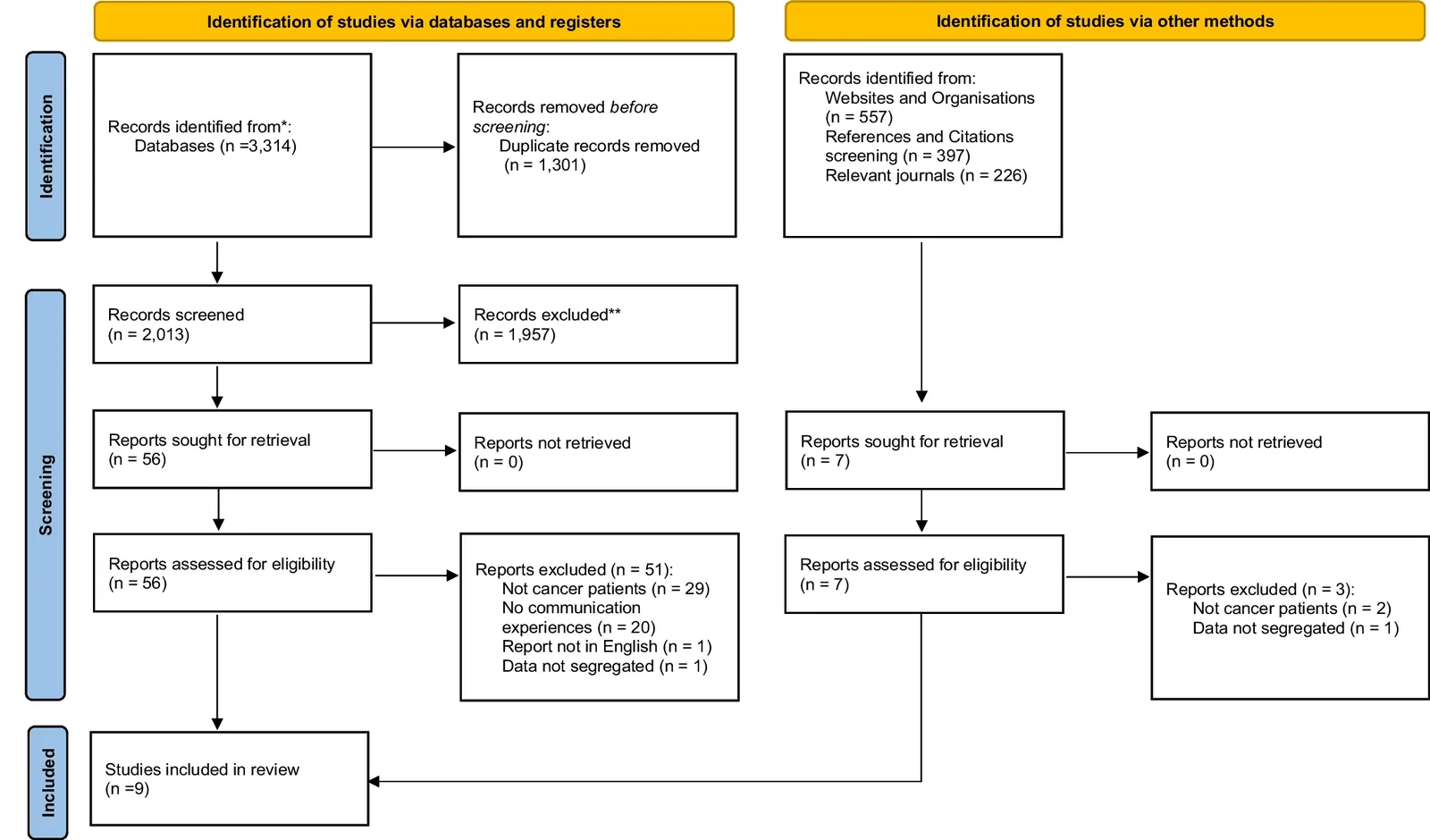
PRISMA flow diagram

The database search yielded 3314 papers, of which 1301 were duplicates and removed (Fig. 1). Following title and abstract screening, 1957 papers were irrelevant and excluded. The remaining 56 papers underwent full-text review, resulting in the inclusion of 5 studies. An additional 1180 papers were identified through grey literature search and reference and citation screening, of which 7 underwent full-text review, and 4 met the inclusion criteria. In total, 9 studies were included, of which 2 reported on the same study sample [36, 37].

### General characteristics of studies

An overview of the descriptive characteristics of the included studies is presented in Table 2. Studies were conducted between 2009 to 2024 in the USA (*n* = 7) [14, 36–41], Israel (*n* = 1) [42] and Germany (*n* = 1) [43]. Sample sizes ranged from single narratives (*n* = 1) to larger cohorts (*n* = 46). Seven studies adopted qualitative methodologies [14, 36–38, 40–42], primarily using personal narratives, interviews and focus groups. One study was quantitative (questionnaire-based) [43], and one employed a mixed-methods design [39].

**Table 2:** Study characteristics

Author, year	Country	Study aim	Study design	Methods	Population	Mode of communication	Peer review
Ackerman 2020 [14]	USA	Understand the experience of a deaf patient with TNBC and interaction with HCPs/interpreters	Qualitative	Personal narrative	1 deaf patient diagnosed with TNBC (age NR, female)	Sign language	No
Bergeron et al. 2024 [38]	USA	Identify barriers and gather recommendations for community support professionals’ training resources	Qualitative	Focus groups using semi-structured questions	20 participants Deaf/HOH cancer patients^a^ (*n* = 8) Advocates/community support professionals (*n* = 6) Mixed hearing/HOH HCPs (*n* = 6) Age NR Gender NR	Sign language	Yes
Berman et al. 2017 [39]	USA	Evaluate and address the breast cancer educational needs of D/deaf patients	Mixed-methods	Interviews + survey	29 deaf patients with breast cancer Interviews *n* = 7 Age 4/7 > 60 years Survey *n* = 22 Age 16/22 > 60 year Gender: female	Sign language	Yes
Feilbach 2023 [41]	USA	Explore HCPs experiences and preferences of working with interpreter teams^b^	Qualitative	Semi-structured interviews using 2 simulated video consultations with interpreters	9 HCPs Age: mean 45 years (range 30–72 years) Gender: NR	N/A	Yes
Faix-Wilkinson 2009 [40]	USA	Understand the experiences of deaf patients in social support groups and the impact of them	Qualitative	Survey: semi-structured; open and closed questions; online/paper (including pilot survey)	53 patients with breast cancer incl. 3 deaf (pilot survey) 20 Deaf patients with breast cancer (main study) Age: NR Gender: female	Sign language	No
Keck et al. 2023 [43]	Germany	Explore deaf cancer patients and their perspective on communication issues with physicians and draw recommendations for physicians	Quantitative	Study-specific + validated questionnaires^c^	104 cancer patients, 46/104 HOH Age: mean 77 years (range 32–90 years) Gender: 47.1% male and 52.9% female	Speech (supported by hearing aids)	Yes
Tannenbaum-Baruchi 2023 [42]	Israel	Explore the needs of patients with hearing disabilities through narrative	Qualitative	Personal narrative	1 deaf patient with Hodgkin lymphoma (aged 75 years, male)	Speech (supported by lip-reading)	Yes
Yabe 2019 [36], 2022 [37]	USA	Explore the experience of D/HH patients who used VRI	Qualitative	Narrative interviews	8 deaf patients, incl. 1 with breast cancer (aged 53 years, female)	Sign language	No

Abbreviations

*CHN*, community health navigators; *D/HH*, Deaf/hard of hearing; *DI-HI*, Deaf-interpreter hearing-interpreter; *HCPs*, healthcare professionals/providers; *HOH*, hard-of-hearing; *NR*, Not reported; *TNBC*, triple negative breast cancer; *VRI*, video remote interpretation

^a^This study included deaf blind cancer patients

^b^Includes deaf and hearing interpreters

^c^WHO (Five) Well-Being Index (WHO-5); Abbreviated Profile of Hearing Aid Benefit (APHAB)s

Eight studies involved d/Deaf cancer patients [14, 36–40, 42, 43]. Five reported on female breast cancer patients [14, 36, 37, 39, 40], one on a male patient with Hodgkin lymphoma [42], one included a mixed gender group [43] and one did not report gender [38]. In two studies, the cancer group was not specified [38, 43]. Only one study focused on HCPs experiences with d/Deaf cancer patients [41]. No papers on CSPs experiences were found.

### Patients’ experiences of communication

The results of thematic analysis are displayed in Table 3, including illustrative quotes with the quote number dispersed throughout. Three main themes and nine subthemes were identified through team discussion. System-level factors included lack of access to interpreting services, unavailability of qualified interpreters and logistical issues. Interpersonal factors related to limited deaf awareness in healthcare, HCPs’ negative attitudes and misconceptions, communication style issues and communication logistics. Finally, patient-level factors included health literacy and understanding, and personal-level barriers.

**Table 3:** Themes and subthemes

Themes	Subthemes	Number of studies	Quotes [quotation number]
**System-level factors**	*Lack of access to interpreting services* Patients frequently reported on the unavailability of interpreters during their consultations	Ackerman [14] Berman [39] Yabe [36, 37] Bergeron [38] 4	“[…] They provided interpreters but sometimes there were no interpreters available or the interpreter scheduling, they had to leave right after the job […]” (Yabe, 2019, page 121) [Q1]
	*Qualified interpreters unavailable* Some patients reported that interpreters were inaccurate in interpreting information, or not medically trained	Ackerman [14] Berman [39] Faix-Wilkinson [40] 3	“[…] I did not trust these interpreters because the interpreter agency hired noncertified people. […] I felt that I would have gotten more detailed information if I have a qualified and certified interpreter present at this medical appointment.” (Ackerman, page 39) [Q2]
	*Logistical issues* Patients experienced inadequate room setup or being unable to request interpreters through portals	Bergeron [38] Keck [43] Ackerman [14] 3	“When I type to book appointments through patient portals, there is no option/way to request interpreters” (Bergeron, page 355) [Q3]
**Interpersonal factors**	*Lack of deaf awareness in healthcare* Patients reported that HCPs lacked understanding of their communication needs and underestimated the importance of interpreters	Berman [39] Ackerman [14] Bergeron [38] Tannenbaum-Baruchi [42] Yabe [36] 5	“My doctor “doesn’t understand deafness” and failed to answer questions, including about cancer because I was D/deaf and they didn’t communicate with me.” (Berman, page 1169) [Q4]
	*HCPs negative attitudes and misconception* Patients experienced negative attitudes from HCPs, e.g. lack of patience or frustration and little effort was made to use their preferred communication method	Tannenbaum-Baruchi [42] Faix-Wilkinson [40] Berman [39] Bergeron [38] Ackerman [14] Keck [43] 6	“Throughout the course of diagnosing, caring for, and treating my father, the health care workers spoke to me, not to my father – the patient- although he was right in front of them. Several spoke to me as if my father were my child or had a cognitive disability. They were even surprised when my father asked a lot of questions about his conditions.” (Tannenbaum-Baruchi, page 536) [Q5]
	*HCPs style of communication* Patients reported that HCPs were insufficient in their communication techniques, including lack of eye contact or speaking too fast	Faix-Wilkinson [40] Keck [43] Bergeron [38] Ackerman [14] 4	“I ask doctors to look at me when they speak or to speak slowly and clearly, but they forget within five minutes. Not their fault. My hearing loss is not their experienced reality.” (Faix-Wilkinson, page 64) [Q6]
	*Logistics of communication* Some patients expressed difficulties in understanding the HCPs when wearing face masks or reported connectivity issues when using VRI	Yabe [37] Keck [43] 2	“I noticed that when you have an in-person interpreter, there is information that you can build upon and retain. However, with VRI, they do not necessarily understand the doctor. As a result, there is a lot of misunderstanding between myself and the interpreter.” (Yabe, 2022, page 32) [Q7]
**Patient-level factors**	*Health literacy and understanding* Some papers highlighted inaccessible information and knowledge gaps that affected patient’s health literacy and understanding	Bergeron [38] Berman [39] Keck [43] 3	“Most common confusion among our deaf community members is thinking that the prostate itself are testicles. I keep telling them, “No, it’s inside the bladder. […] They often say, “Oh, I didn’t realize.” […] (Bergeron, page 357) [Q8]
	*Personal-level barriers* Patients reported feelings of distrust and disconnection with HCPs	Berman [39] Keck [43] Faix-Wilkinson [40] Bergeron [38] 4	“I respected my doctor but not always trusted him full […]” (Faix-Wilkinson, page 79) [Q9]

Abbreviations

*HCPs*, healthcare professionals; *VRI*, video remote interpretation

#### System-level factors

Many systemic barriers are faced by d/Deaf people when accessing healthcare, which may compromise the quality of care and support they receive. A reoccurring concern identified across multiple papers was the lack of interpreters during consultations [14] [36–39] [Q1]. This included conversations where the cancer diagnosis was shared [39] and could contribute to difficulties understanding the disease and treatment process [38–40, 43]. Feelings of loneliness, isolation or frustration were often reported as a result [14, 40].

In contrast, the presence of a trained interpreter appeared to improve the patients’ experience and understanding [14, 40]. One patient shared:

The first doctor wrote back and forth, then we found interpreters, which was more helpful [40] (page 81).

However, concerns about interpreter quality were highlighted in several studies (*n* = 3) [14, 18, 40] with reports that some lacked medical training or misinterpreted information which could have affected the patient’s treatment decisions and confidence in interpreting services [14, 39][Q2]. One patient shared:

One of the medical professionals said that there were high risks, the interpreter interpreted in ASL, “DANGEROUS DEAD CAN!”, which means in English, “It is dangerous, and you can die!”. So, based on the interpretation, it impacted my decisions. If the interpreter interpreted this properly, I would have made different decisions. [14] (page 64)

To address these issues, some studies (*n* = 2) [14, 39] recommended employing medically trained interpreters to reduce miscommunication and maintaining interpreter consistency to support continuity of care. Additionally, allowing more time for consultations could also facilitate clearer communication [43].

Another challenge identified was that medical terminologies and concepts were not always accessible in sign language [38], which may have affected comprehension. There were also logistical issues identified. These included poorly designed rooms that obstructed the patients’ view of the interpreter [14] and being unable to request interpreters through patient portal [38] [Q3]. These systemic barriers may negatively influence patients’ understanding of their cancer diagnosis and treatment options and could reduce their trust in the healthcare system. One patient commented:

I don’t trust them [HCPs]. They don’t tell me the truth as it is, so I am my own doctor. I do my own research. I am sick of doctors trying to coerce or rush me into a decision making on the spur of the moment or won’t give me the time of day [40] (page 79)

#### Interpersonal factors

There were various interpersonal factors that shaped the challenges d/Deaf patients face throughout their cancer care and may have influenced patient-provider interactions. One recurring theme was the lack of deaf awareness within healthcare settings. Many papers reported that HCPs had limited understanding of d/Deaf culture and their communication needs [Q4], with some HCPs relying on inadequate alternatives like lipreading or written notes [14, 18, 40]. One patient shared:

It was awful because no one would try to communicate with me and [I] couldn’t read their lips. I felt like I wasn’t treated like a human. [39] (page 1171)

HCPs’ negative attitudes were also reported, with patients describing impatience, frustration or reluctance to adapt communication to the needs of the patient [14, 39, 40] or not allowing sufficient consultation time [43].

A few papers revealed that some HCPs held misconceptions about d/Deaf people, believing that patients did not require an interpreter since they knew how to lipread [14, 39] or assuming patients were illiterate. One paper described that this almost led to exclusion of the patient from a clinical trial [42] [Q5]. To address these issues, several studies recommended targeted training to improve d/Deaf cultural competence and awareness for HCPs and healthcare staff to assist in promoting accessible care [14, 36, 38, 42].

Another challenge identified was the way HCPs communicated with patients, which may have influenced how information was perceived and interpreted. Patients frequently described poor communication practices from HCPs [Q6]. A study by Keck et al. [43] showed that in a study-specific survey 87% of patients found that HCPs spoke too quickly, while 43% reported that eye contact was not maintained. The use of face masks further posed a challenge, with 85% stating that it obscured facial expressions which may have hindered communication. Technical issues such as poor connectivity also disrupted effective communication when using video remote interpreting (VRI) services [36, 37] [Q7]. These challenges could have impacted patients’ understanding of cancer treatment and their decision-making. It was also reported that over a third of the respondents reported difficulty understanding the full scope of treatment and making decisions about treatment options based on the information provided during informed consent conversations [43]. To improve communication, Keck et al. [43] recommended that HCPs speak clearly and maintain eye contact when interacting with patients.

#### Patient-level factors

Some d/Deaf patients described that due to inaccessible health communication they experienced difficulties in understanding and processing information throughout their care. Although physicians, family and friends were identified as the main sources of health information [39], these sources appeared to be inadequate. For example, Berman et al. [39] described breast cancer survivors who lacked understanding of what cancer was and were uncertain about the procedures and medication they had received. Similar findings were noted by Keck et al. [43], who reported that 72% of d/Deaf patients struggled to understand verbal instructions about medication and examinations, highlighting a critical gap in accessible communication and information [Q8]. The use of visual cues or written materials was suggested as a strategy that might improve the understanding of procedures [14]. Some patients also described personal barriers to communication, like discomfort to ask for clarification. One patient commented:

a lot of D/deaf people won’t stand for themselves. They think they don’t need to communicate. They are suffering silently… I think the D/deaf people let things stop them from what they want to do [39] (page 1172)

Additionally, four studies [18, 38, 40, 43] reported that d/Deaf patients often felt disconnected from HCPs, with some expressing distrust which could be associated with previous negative experiences [40][Q9].

### HCPs experiences of communication with d/Deaf cancer patients

Only one paper [41] reported on the experiences of HCPs communicating with d/Deaf cancer patients. This study explored HCPs’ perceptions and preferences on working with interpreter teams, using simulated videos and semi-structured interviews. HCPs reported experiencing improved communication and patient interaction in presence of interpreters and felt more enabled to evaluate and assess patients. One HCP described how important information can sometimes become lost without the use of interpreters:

I spoke to the patient in their primary language [American Sign Language] using an interpreter for the first time since they’ve been here, and they have no clue that they have cancer […] Like, it’s happened multiple times. [41] (page 14)

The study highlighted the importance of understanding why interpreters are essential for d/Deaf patient’s understanding of their condition, and how they may foster a positive patient experience. It was suggested by HCPs that this could be an opportunity for further education on the benefits of interpreting services and deaf culture.

## Discussion

This scoping review is the first to systematically examine the experiences and unmet needs of d/Deaf patients within cancer care. The findings revealed a range of barriers that could have disrupted effective communication and shaped patients’ perceptions of care. Communication was frequently undermined by the lack of interpreting services, absence of qualified medical interpreters and inadequate room configurations affecting patient-provider interactions. The literature also highlighted an absence of Certified Deaf Interpreters (CDIs). CDIs are deaf or hard-of-hearing interpreters whose cultural knowledge, lived experience and specialised training enable them to work effectively with hearing interpreters and reduce communication barriers through a range of techniques, such as gestures, mime, props and other tools. [44]. Their involvement can be particularly important for complex medical appointments where their combined linguistic and cultural expertise can enhance the accuracy and effectiveness of communication.

Patients also reported that HCPs demonstrated limited understanding of d/Deaf culture, which may have contributed to negative or dismissive interactions that could have fostered distrust and disconnection towards HCPs. In addition, communication practices such as rapid speech and lack of eye contact reduced the patients’ ability to understand medical information. Similar challenges were observed in video remote interpretation services, where poor connectivity restricted comprehension. Collectively, these barriers reflect the broader consequences of inaccessible healthcare and the systemic exclusion of d/Deaf individuals. Evidence from SignHealth, a UK-based deaf health charity, highlights the extent of these inequalities; only 11% of Deaf patients reported having equal access to NHS services, while 81% stated that their communication needs were not adequately met during healthcare appointments [45]. As a result, many patients experienced challenges understanding their diagnosis, treatment options and the information necessary to make informed decisions about their care. One patient noted:

Well, you know maybe if you went to a family doctor, family practice for maybe a cold or a sore throat, bronchitis, something not as complex, I probably would not mind it so much. But for the type of situation [cancer treatment] I was experiencing, that was not acceptable. [36] (page 105)

Similar communication challenges have been documented in other healthcare settings. Parmar et al. found in a survey among 556 d/Deaf patients and informal caregivers that communication needs were often not being met during HCP appointments [46]. Many reported experiencing emotional fatigue due to the effort required to engage in communication, with around two-thirds indicating that they missed 50% or more of the information provided. Only 32% expressed satisfaction with their HCPs’ communication skills. These findings were also reflected in a study looking at cancer care experiences among patients with other disabilities, such as physical, cognitive, sensory and mental-health conditions [47]. This is consistent with the social model of disability, which argues that disabilities arise when environments and systems fail to accommodate the individuals’ needs, with participants emphasising the importance of an integrated, person-centred approach to ensure equitable care [48].

Evidence exploring the communication experiences of HCPs and CSPs working with d/Deaf cancer patients is lacking. This review only included one study, which suggested that HCPs may benefit from additional education and training about the d/Deaf community to facilitate better interactions and reduce the likelihood of miscommunication. Cultural competency programs like the Deaf Strong Hospital, an educational program that exposes medical students to communication, linguistic and cultural issues that are relevant to providing effective patient care for d/Deaf patients [49], have demonstrated improvements in students interacting with these patients by increasing awareness of communication preferences. Other training programs have similarly shown positive effects on nurses’ self-efficacy [50].

### Strengths and limitations

A strength of this review is that it provides a detailed overview of the communication experiences of d/Deaf cancer patients in healthcare and mapped their unmet needs. The inclusion of grey literature featured rich patient-centred narratives. The review findings could be used to provide recommendations for clinical practice and future research.

There are several limitations to this review. The evidence was largely exploratory and limited by smaller sample sizes; as such the findings were interpreted tentatively. The evidence base was also narrow and disproportionately focused on women with breast cancer (typically supported by more established care pathways), limiting the transferability to other cancers, particularly rarer or less-resourced conditions. Additionally, there was limited involvement of deaf community members in the design and interpretation of the included studies. All studies were published in English, which may have excluded relevant research published in other languages. The majority (78%) of the studies originated from the USA, limiting the generalisability of the findings to other healthcare systems. Only one study focused on HCPs’ communication experiences and none on CSPs, leaving major gaps in understanding their perspectives and educational needs.

### Future research directions and practical implications

The results of this scoping review may suggest several opportunities for further research. Evidence suggests that some d/Deaf individuals may experience barriers to accessing or engaging with healthcare information, including challenges related to health literacy. A systematic review by Choi et al. found positive results from health (mainly cancer) educational interventions for people with hearing loss, with video-based materials appearing to have improved knowledge [51]. More recently, a review by Münstermann et al. [52] demonstrated that individualised educational programs may further enhance cancer-related health literacy among d/Deaf people. Other programs have demonstrated similar benefits, including increased knowledge about early detection in prostate cancer [53] and breast cancer or cervical cancer screening practices [54, 55], with knowledge retention lasting up to two months. However, further research is required to understand the long-term impacts of these programs.

Deaf awareness training could positively influence HCPs’ interactions with the d/Deaf community. However, recent systematic review found that such training is limited to small groups of health professional students in only six countries and typically occurs during their initial training [56]. This highlights the need for broader implementation and integration into healthcare learning platforms. Involving facilitators with lived experience is essential to ensure the training reflects the realities of d/Deaf peoples’ experiences. System-level improvements are also required to ensure reliable access to qualified medical interpreters, which is necessary for equitable cancer communication to d/Deaf patients.

Further research should also explore the perceptions of CSPs during cancer consultations, particularly around the challenges they encounter within the healthcare setting. This is especially significant given that cancer diagnosis and treatment have become increasingly complex, placing greater communication demands on CSPs. Evidence from medical interpreters working with cancer patients with limited English proficiency showed that targeted cancer-specific training was associated with a 23% improvement in their knowledge about cancer [57]. Developing similar training for CSPs could strengthen their understanding of common cancer terminology and may enhance the accuracy and clarity of communication between d/Deaf patients and HCPs. Currently, a survey to assess the educational needs of CSPs is underway (https://shore-c.sussex.ac.uk/study.php?projid=207) to help identify training and resources that may be required to enable CSPs to support d/Deaf patients more effectively.

## Conclusion

Despite limited data, this review highlights the wide range of barriers d/Deaf cancer patients can encounter when accessing cancer services. Collectively, these barriers could affect effective communication practices, compromising information provision, comprehension and the quality of care delivered. The existing evidence supports the use of qualified medical interpreters, accessible, visually based educational interventions to improve health literacy among d/Deaf individuals and the rollout of deaf awareness training for HCPs.

## Supplementary Information

Below is the link to the electronic supplementary material.

ESM 1 (DOCX 15.2 KB)

## Data Availability

No datasets were generated or analyzed during the current study.

## Declarations

### Patient and public involvement

Patients or members of the public were not involved in the design, conduct and reporting of this scoping review.
