# Supplementary material for: Communication barriers for d/Deaf people with cancer: a scoping review
Source: Support Care Cancer. 2026 Sep 9;34(10):950. doi: 10.1007/s00520-026-11202-y (PMC13558441; doi:10.1007/s00520-026-11202-y)
Supplement: Supplementary file 1 [file 520_2026_11202_MOESM1_ESM.docx]

**Supplementary file 1: Search strategy developed for PubMed and adjusted for other databases**

1. What are d/Deaf cancer patients’ communication experiences and unmet needs in healthcare settings?

(deaf OR "hard of hearing" OR deafblind) AND cancer AND healthcare AND communication AND experiences

2. What are the experiences and educational needs of healthcare professionals working with d/Deaf cancer patients?

("healthcare professional*" OR nurse OR physician*) AND (deaf OR "hard of hearing" OR deafblind) AND cancer

3. What are the experiences and educational needs of communication support professionals working with d/Deaf cancer patients in healthcare settings?

experience* AND ("communication professional" OR signers OR interpreter*) AND (deaf OR "hard of hearing" OR deafblind) AND cancer
